# Primary plasma cell leukemia presenting as a thoracic mass

**DOI:** 10.11604/pamj.2014.19.39.4546

**Published:** 2014-09-18

**Authors:** Marielle Igala, Regis Gothar Bopaka, Wiam Khtabi, Said Benchekroun, Asma Quessar

**Affiliations:** 1 Hematology Department, CHU IBN Rochd, Faculty of Medicine and Pharmacy, Universite Ain Chock, Casablanca Morocco; 2 Pneumology Department, CHU IBN Rochd, Faculty of Medicine and Pharmacy, Universite Ain Chock, Casablanca Morocco

**Keywords:** Plasma cells leukaemia, thoracic mass, pulmonary embolism

## Abstract

Primary Plasma cell leukaemia (pPCL) is a rare plasma cell (PC) malignancy. The strict criteria for the diagnosis is an absolute PC number greater 2 X 10^9^/L or a plasmocytosis accounting for > 20% of the differential white cell count that does not arise from a pre-existing multiple myeloma. pPCL was associated with aggressive clinic-biological features. Primary Plasma cell leukaemia is more characterised by an extra medullar involvement such as hepatomegaly, splenomegaly, lymphadenopathy, lepto-meningeal infiltration or extramedullary plasmocytomas. The prognosis of pPCL is very poor. We report the case of a fifty eight year-old man directed to the haematology department for diagnosis of pPCL revealed by a thoracic plasmocytomas mimicking a thoracic neoplasm. The patient received chemotherapy including a classic treatment for multiple myeloma but developed a pulmonary embolism. This case illustrates an uncommon presentation of pPCL the difficulty treating by multiple myeloma chemotherapy.

## Introduction

The term plasma cell leukaemia (PCL) is usually used when the number of circulating plasma cells is significant. It's the most aggressive form of the plasma cell dyscrasias. It is defined by the presence of > 2 X 10^9^/L peripheral blood plasma cells or accounting for >20% of the differential white cell count, that does not arise from pre-existing multiple myeloma (MM). Secondary PCL is a leukemic transformation of end-stage MM. PCL is rare, with only 1-4% of MM patients presenting as PCL. In addition, < 1% of patients presenting with extreme leucocytosis (>50 X 10^9^/L) are diagnosed with PCL. Extramedullary involvements, such as hepatomegaly, splenomegaly, lymphadenopathy, leptomeningeal infiltration, or extramedullary plasmocytomas, are more frequent in PCL. The prognosis of PCL is very poor, with a median overall survival of only 7 months with standard chemotherapy. [1–3] We report the case of a fifty eight year-old smoker male with a diagnosis of PCL which presented as a thoracic mass which was barely treated.

## Patient and observation

**Figure 1:**
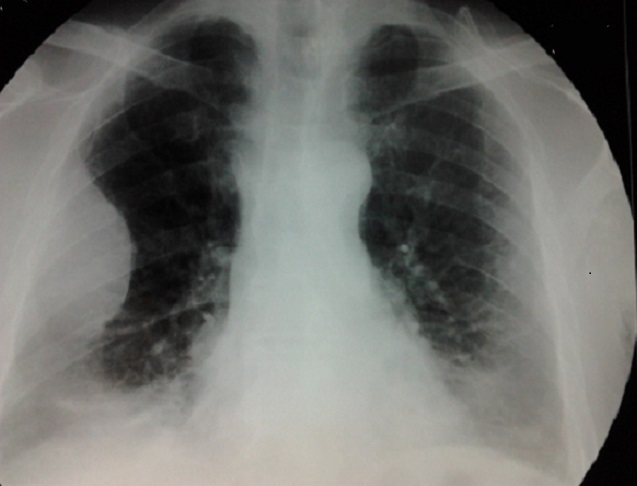
Chest x ray revealed a right opacity homogenous well limited

**Figure 2:**
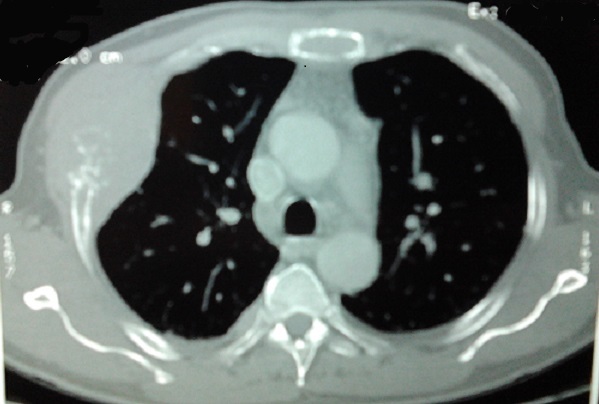
Chest computed tomography revealed right mass with lysed costal lesion

**Figure 3:**
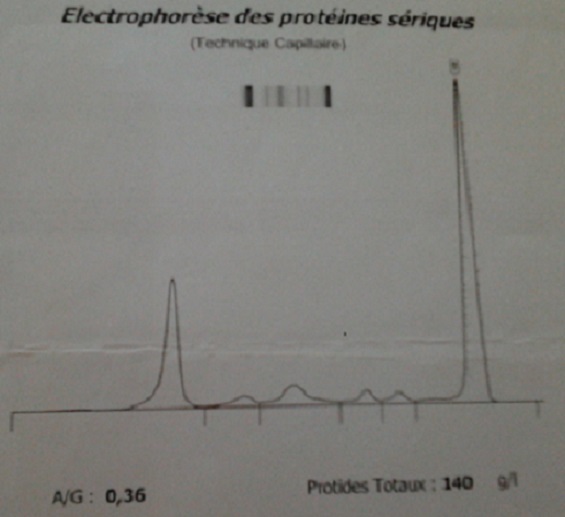
Protein electrophoresis showed a Gamma-globulin peak

A 58-year-old smoker male without past medical history was referred to our hospital with over three months’ right chest and para-sternal pain. Physical examination found a performance status at 2, a dyspnea, a cough with hemoptisis, and painful right chest and para-sternal mass without fever or weight loss. The chest x ray revealed the presence of a right pulmonary opacity (Figure 1) and chest computed tomography a large parietal mass of the 4th costal arch of 95 X 45 mm with lysed appearence of the manubrium and sternal body, and a pathological fracture of the 5th right rib. (Figure 2) A transmural ultrasound biopsy of the mass allowed the detection of a malignant undifferentiated tumour growth with a strong positivity of CD138 and anti Kappa antibodies. Laboratory evaluation showed a white blood cell count of 22 × 10^9^/L with 58% of plasma cells, haemoglobin of 8.6 g / dl, normal platelet count. The peripheral blood smear showed red blood cell rolls and an atypical appearance of white blood cells. The blood chemistry found a creatinin at 15.6 mg /L (reference range < 12 mg/L) urea at 0.71 g /L (reference range < 0.4 g/L) and calcium at 100mg/L. Electrophoresis of blood and urine proteins showed respectively the presence of a monoclonal peak of gamma globulin 79.5 g/L (Figure 3) and Bence Jones protein free Kappa. The bone marrow biopsy demonstrated diffuse infiltration with 96% of atypical plasma cells. On the skull x-ray lytic lesions were visible. The patient was referred to the haematology department where the examination was supplemented by flow cytometry on peripheral blood which showed 48% of plasma cell population with Kappa monotypic antigen, positivity to CD38 + /CD138+ at 98%, CD19+ and CD56+. CD20 and CD117 antigen were not expressed. The cytogenetic examination of bone was a failure. Treatment according to the protocol Cyclophosphamide, Thalidomide and Dexamethasone was started but a rapid deterioration of the clinical appearance of the patient was observed, followed by a pulmonary embolism.

## Discussion

As our clinical case, plasma cell leukaemia's patients have a younger age at diagnosis (range from 40 to 62 years) compared with MM or secondary PCL patients. Their performance status at diagnosis is usually worse, which may be related to the more advanced stage of disease. [1, 2] Plasma cell leukaemia is characterised by a diverse nonspecific presentation which is not suspect to be plasma cell dyscrasia. Rakhee Kar in the study of five patients with pPCL found fatigability, loss of appetite, fever, abdominal distension and pedal edema. He also found the duration of illness ranged from one week to two months. [3] Extramedullary presentations such as hepatomegaly, splenomegaly, lymphadenomegaly, leptomeningeal infiltration or extramedullary plasmocytomas are more frequent in pPCL (23%) than in MM (only 4%). Garcia Sanz et al had in her study subcutaneosus nodes, peritoneal plasmocytomas meningeal infiltration and parapleural mass. [2] Osteolytic lesions have a lower prevalence than in MM. This can be explained by the fact that pPCL cells leave the bone marrow and enter the peripheral circulation before forming bone tumors, presumably because of a loss of homing signals in the bone marrow [1, 4]. Our patient had thoracic pain and tumour which imitated an extensive chest bone neoplasia. Tumour investigation found an infiltration by plasma cells with peripheral blood involvement.

Peripheral blood smear found an atypical appearance of white blood cells. Flow cytometry in plasma cell leukaemia usually express as MM CD38 and CD138. However there is a reduced expression of CD56, CD117, CD71 and HLA-DR antigens compared to MM. Plasma cell leukaemia is more likely to express CD20, CD45, CD19, CD27 and CD23. [1–3, 5] In our case besides antigens usually found in plasma cells expressly CD56 and CD19. Our patient didn't have a cytogenetic analysis because of its failure. The value of additional risk stratification by cytogenetic to guide the type of therapy or predict outcome is currently limited in primary plasma cell leukaemia because it is a clearly defined high-risk plasma cell disorder. Chromosome 13 abnormality is known to have a high incidence in primary plasma cell leukaemia [1–3]. Primary plasma cell leukaemia requires urgent control of clinical manifestations to prevent early death because of irreversible disease complications but its treatment modality is not well known. No randomized trials have been reported exclusively for these patients. The prognosis after conventional chemotherapy without novel agents such as bortezomib, lenalidomide and thalidomide is poor with an overall survival of 7 months. [1, 6, 7] Our patient received thalidomide but quickly developed a pulmonary embolism and decrease of his clinical appearance after only 1 month of treatment.

## Conclusion

This case highlights that primary plasma cell leukaemia, a rare plasma cell dyscrasia, can have an uncommon localization. The prognostic of this disease is poor with a bad treatment response despite novel chemotherapy drugs.

## References

[1] Van de Donk NWCJ, Lokhorst HM, Anderson KC, Richardson PG (2012). How I treat plasma cell leukemia. Blood.

[2] Garcia-Sanz R, Orfao A, Gonzalez M, Tabernero MD, Blade J, Moro MJ (1999). Primary Plasma Cell: clinical, Immunophenotypic, DNA ploidy, and cytogenetic characteristics. Blood.

[3] Kar R, Priyadarshmini SG, Niraimathi M, Basu D, Badhe BA (2012). Clinico-pathological spectrum of primary plasma cell leukemia diagnosed at a tertiary care centre in south Indian over 5 year period. Indian J Hematol Blood Transf..

[4] Talamo G, Dolloff NG, Sharma K, Zhu J, Malysz J (2012). Clinical features and outcomes of plasma cell leukemia: a single-institution experience in the era of novel agents. Rare Tumors..

[5] Tembhane PR, Subramanian PG, Seghal K, Yajamanam B, Kumar A, Gadge V (2011). Immunophenotypic profile of plasma cell leukemia: a retrospective study in a reference cancer center in indian and review of literature. Pathology et microbiology.

[6] Rodriguez C, Pont JC, Gouin-Thibault I (2005). La leucémie a plasmocytes. Ann Biol Clin..

[7] Avet-Loiseau H, Daviet A, Brigaudeau C, Callet-Bauchu E, Terre C, Lafage-Pochitaloff M (2001). Cytogenetic, interphase and multicolor fluorescence in situ hybridization analyses in primary plasma cell leukemia: a study of 40 patients at diagnosis, on belhaf of the Intergroupe Francophone du Myelome and the Groupe Francais de Cytognetique Hematologique. Blood.

